# 

**Published:** 1901-08

**Authors:** 


### ASSOCIATION MEETINGS.

### AUGUST.

### ILLINOIS VETERINARY MEDICAL AND SURGICAL ASSOCIATION.

THE twelfth semi-annual meeting of the Illinois Veterinary Medical and Surgical Association will be held at Decatur, Ill., on Thursday and Friday, August 8 and 9, 1901.

The programme includes :

*Thursday*, August 8, 1901—The President's address; order of business; "Lymphangitis," by Dr. N. P. Whitmer, Gardner; "Catarrhal Fever," by Dr. C. A. Hurlbutt, Stonington; "Fistula," by Dr. W. J. Martin, Kankakee; discussion of papers; reports of cases.

*Friday*, August 9, 1901—"Osteoporosis," by Dr. S. H. Swain, Decatur; "Anthrax," by Dr. V. G. Hunt, Arcola; "Tetanus," by Dr. R. W. Braithwaite, Champaign; "Laminitis," by Dr. S. D. Brown, Assumption.

A large attendance is expected.

### NEW HAMPSHIRE VETERINARY MEDICAL ASSOCIATION.

The regular meeting of the New Hampshire Veterinary Medical Association will be held on Friday, August 2, 1901.

### NIAGARA AND ORLEANS COUNTY VETERINARY MEDICAL SOCIETY.

The Niagara and Orleans County Veterinary Medical Association (New York State) will hold its annual meeting at Lockport, New York, on August 16th.

There will be an election of officers and reading of papers by the President, Dr. W. E. Stocking, of Medina; Dr. Mark I. Williams, of Middleport, N. Y.; Dr. J. O. Moore, of Wilson, N. Y., and others.

### SEPTEMBER.

### A. V. M. A.

Atlantic City, September 3d, 4th, and 5th. See the letter of the Secretary, Dr. S. Stewart, on page 528 of this number of the JOURNAL.

### INDIANA STATE VETERINARY ASSOCIATION.

In Indianapolis in September. Date and programme will be given in the September JOURNAL.

### SOCIETY OF VETERINARY GRADUATES OF WISCONSIN.

The semi-annual meeting of the Society of Veterinary Graduates of Wisconsin will be held in Milwaukee during the second week of September. The exact date and programme will be published in the September JOURNAL.

### PENNSYLVANIA STATE VETERINARY MEDICAL ASSOCIATION.

The semi-annual meeting will be held in Pittsburg, September 17th. Programme later.

### NEW YORK STATE VETERINARY MEDICAL SOCIETY.

The annual meeting will be held at Ithaca on September 10th and 11th.

Papers have been announced as follows: "Metritis," by J. A. Bell, of Watertown; "Supernumerary Digit in a Foal," by J. L. Wilder, of Akron; "Fracture of Ribs, with Protrusion of Omentum," by W. H. Salisbury, of Clifton Springs; "The General Utility Horse" (illustrated), by C. D. Morris, of Binghamton; "Specific Etiology," by V. A. Moore; "Retained Placenta," by W. L. Williams; "Multiple Tumors in the Pig," by V. A. Moore and S. H. Bennett; "Spaying as a Remedy for Vice in the Mare," by A. H. Ide, of Lowville. Other papers by E. B. Ackerman, George H. Berns, James Law, and P. A. Fish.

An extensive clinical programme has been provided.

Important business and serious matters concerning the welfare of the Society will come under discussion. A large and brilliant meeting is expected.

For information as to reduced railroad fares and hotel accommodations, write to Dr. W. L. Williams, Ithaca, New York.

---

*Veterinary Register.* The publication of the *Veterinary Register* in the JOURNAL will be commenced in the September number. Veterinarians in Pennsylvania and Ohio who have not returned their names with correct address are requested to do so at once. The blanks for veterinarians in other States will be sent out shortly.

### PERSONALS.

President William H. Lowe, of Paterson, N. J., was a very happy man on the day of the annual meeting of their State Association on receiving from his colleagues in the State a handsome gavel, on the gold band of which was inscribed a suitable reminder of the source of this much-prized token of recognition.

Dr. Leonard Pearson sailed for Europe on July 31st. He will be absent six weeks. It is understood that he desires to inspect new veterinary buildings at the colleges abroad, in view of the rebuilding of the Veterinary Department of the University of Pennsylvania.

Dr. T. E. Smith, of Jersey City, is one of the most active spirits in the Road Drivers' Association of that city, and looks forward to an early enactment of the State Legislature in providing a speedway for his city.

Dr. William L. Nunan, of Philadelphia, a graduate of the American Veterinary College, and also in human medicine, takes an active interest in Democratic politics in the "City of Brotherly Love."

Dr. William Steile, of Stratford, Ontario, Vice-President of the Ontario Veterinary Medical Association, is the owner of two well-known thoroughbred stallions.

Ground will soon be broken for the new $25,000 veterinary building at the University Experiment Station. Dr. Reynolds is happy, of course.

Dr. W. L. Zuill, of Philadelphia, has ceased the general practice of medicine, and is now devoting his entire time and attention as an oculist. He is an assistant surgeon at the Wills Eye Hospital.

Dr. J. O. George, of Camden, N. J., is an expert surf bather, and never loses an opportunity of a plunge in the ocean surf when on the Atlantic coast.

Dr. M. V. Miles, of Charleston, Ill., takes an active interest in the Coles County Agricultural Society.

Dr. J. F. Jones, formerly of Marietta, Ohio, is now a resident of Newark, Ohio, but is no longer in active practice.

Dr. John J. Maher, of Philadelphia, has added a canine and feline cemetery to the attractions of his veterinary hospital.

